# Radiation-Related Fractures after Radical Radiotherapy for Cervical and Endometrial Cancers: Are There Any Differences?

**DOI:** 10.3390/diagnostics14080810

**Published:** 2024-04-12

**Authors:** Hana Malikova, Katarina Nadova, Klaudia Reginacova, Karin Kremenova, Lukas Rob

**Affiliations:** 1 Department of Radiology and Nuclear Medicine, Third Faculty of Medicine, Charles University, Faculty Hospital Kralovske Vinohrady, 11000 Prague, Czech Republic; katarina.nadova@fnkv.cz (K.N.); karin.kremenova@fnkv.cz (K.K.); 2 Institute of Anatomy, Second Faculty of Medicine, Charles University, 11000 Prague, Czech Republic; 3 Department of Oncology, Third Faculty of Medicine, Charles University, Faculty Hospital Kralovske Vinohrady, 11000 Prague, Czech Republic; klaudia.reginacova@fnkv.cz; 4 Department of Obstetrics and Gynaecology, Third Faculty of Medicine, Charles University, Faculty Hospital Kralovske Vinohrady, 11000 Prague, Czech Republic; lukas.rob@fnkv.cz

**Keywords:** computed tomography, bone density, age, body mass index

## Abstract

In this study, we reviewed CT/MRI scans and studied the rates of radiation-related fractures in subjects treated for cervical cancer (CC, 63 subjects) by radical radiotherapy (RT) and in subjects treated for endometrial cancer (EC, 64 subjects) by radical surgery and RT. The differences between bone density measured in L1 on pretreatment CT, age and body mass index (BMI) were evaluated. Despite significant differences in RT total dose, age, BMI, etc., between both groups, the rate of radiation-related fractures was similar: 28.6% of CC versus 26.6% of EC subjects. CC subjects with fractures were significantly older (62.4 ± 10.1 vs. 49.0 ± 12.4 years; *p* < 0.001), and their bone densities were significantly lower (106.3 ± 40.0 vs. 168.2 ± 49.5 HU; *p* < 0.001); no difference in BMI was found. EC subjects with fractures were without significant difference in age but had significantly lower bone densities (103.8 ± 29.0 vs. 133.8 ± 42.3 HU; *p* = 0.009) and BMIs (26.1 ± 4.9 vs. 31.8 ± 6.9 kg/m^2^; *p* = 0.003). Bone density strongly correlated with age (r = −0.755) only in CC subjects. Subjects with fractures from both groups had similarly low bone densities (106.3 ± 40.0 vs. 103.8 ± 29.0 HU; *p* = 0.829); however, no correlation between bone density and BMI was found. The rate of radiation-related fractures in both groups was clearly associated only with low pretreatment bone density, reflecting osteoporosis.

## 1. Introduction

Cervical and endometrial cancers are relatively frequent oncological diseases. Cervical cancer is the fourth most common female cancer and fourth most common leading cause of death [1,2]. Human papillomavirus (HPV) is etiologically involved in approximately 95% of cervical cancers. Currently, more than 200 types of HPV are known; however, only 20 types of HPV are responsible for cervical cancer [3]. Endometrial cancer is the sixth most common cancer worldwide [1,4]. Endometrial cancer is a strongly estrogen-dependent tumor. Body mass index (BMI) and waist-to-hip ratio correlate with estrogen level and are associated with increased endometrial cancer risk [5]. Other risk factors included parity, oral contraceptive use, smoking, age of menarche and diabetes [5]. In both diseases, there are large geographic differences in morbidity and mortality according to the economic status of a country. The declining incidence and mortality of cervical cancer in high-income countries is due to access to prevention and treatment [2]. On the contrary, the incidence of endometrial cancer in high-income countries has been growing due to high rates of obesity and physical inactivity in the aging female population [4].

Radical radiotherapy (RT) and concomitant chemotherapy is the preferable treatment option for advanced cervical cancer [6]. Patients suffering from endometrial cancer at higher stages or with risk factors are preferably treated by radical surgery followed by postoperative RT and/or chemotherapy [4]. RT for gynecological cancers generally consists of external beam radiation (EBRT) and brachytherapy (BRT). It is known that radical RT brings a wide spectrum of radiation-related side effects [7,8,9,10,11]. Bone marrow, the urinary bladder and bowels are most commonly affected. Radiation-related proctitis, colitis, enteritis and cystitis are very frequent comorbidities. However, after radical RT, some serious side effects can develop. Ureteral strictures resulting in hydronephrosis, different fistulas, bowel strictures resulting in ileus or bowel perforation, or even secondary tumors or life-threatening vascular complications can be found [7,8,9,10,11]. Some radiation-related side effects can affect treated patients their entire lives [7,8,9,10,11]. Bone marrow is a highly radiosensitive tissue, and the fatty replacement of bone marrow is an inevitable result of RT. Therefore, it is not surprising that radiation-related bone toxicities such as pelvic insufficiency fractures or osteonecrosis are among the most common radiation-related side effects and may be found at any time interval post-irradiation [7,12,13,14,15]. The incidence of pelvic insufficiency fractures or osteonecrosis as the most common adverse effect in bone is not clearly known and varies remarkably among studies; however, most studies report an incidence between 8 and 32% [16,17].

In the related literature, most studies are aimed at patients with cervical cancer; however, patients treated for endometrial cancer have received little interest, and our study tried to fill that gap. Moreover, we hypothesized that females treated for endometrial cancer are at higher risk of radiation-related fractures due to higher age. Therefore, the aim of our study was first to evaluate differences between the rates of radiation-related bone side effects in subjects treated for cervical and for endometrial cancer. The second aim of our research was to evaluate differences between pretreatment variables such as bone density of the L1 vertebral body, patient age and BMI and their relations to radiation-related adverse bone effects.

## 2. Materials and Methods

### 2.1. Patient Selection

We searched the institutional information system and Oncology Department internal database (Faculty Hospital Kralovske Vinohrady, Prague, Czech Rep.) and selected all females treated by radical RT for advanced cervical carcinoma and patients with endometrial carcinoma treated by RT between January 2017 and January 2022. From the patients treated by RT for endometrial cancer, we excluded those who underwent only palliative RT for advanced or metastatic endometrial carcinoma and selected only those who underwent radical surgery followed by RT. We then selected patients with available cross-sectional imaging follow-up more than 6 months from RT, including computed tomography (CT), magnetic resonance imaging (MRI) and positron emission tomography/computed tomography (PET/CT). A six-month time interval from RT was chosen; thus, it is considered as a time limit of expected acute radiation-related common changes and/or acute radiation-related adverse events [12]. Patients who were followed only by ultrasound or X-rays were excluded. Moreover, all available medical records were reviewed. We collected all relevant available clinical data and searched for history of previous or current trauma and history of osteoporosis treatment or long-term corticosteroid therapy, and those were considered as exclusion criteria. We calculated the total received radiation dose as the sum of doses (EBRT, BRT and lymph node radiation). Temporal data related to cross-sectional imaging and number and type of imaging examinations were collected as well. We calculated BMI from patient height and weight at the time of diagnosis. 

The selected subjects were divided into two groups according to the main diagnosis: the CC group, patients treated for cervical carcinoma; the EC group, patients treated for endometrial carcinoma. Patients in the CC group were treated by radical chemo-radiotherapy that consisted of pelvic EBRT with the upper edge of the radiation field at the level of the L5 vertebra and by intracavital BRT. All patients in the EC group underwent radical surgery that was followed by pelvic EBRT and vaginal BRT. Some patients from both groups underwent lymph node RT at variable doses. 

The study had a retrospective design, was performed in accordance with Declaration of Helsinki and was approved by the local ethics committee. All patients provided signed, informed consent to participate in the study. 

### 2.2. Bone Density Measurement and Cross-Sectional Imaging Analysis

All patients underwent native abdominal and pelvic CT imaging for RT purposes. All CT examinations were performed on a 128-slice CT scanner (Definition AS+, Siemens Healthineers, Erlangen, Germany) with the following parameters: rotation time 0.5 s, kilovoltage peak 120 kV, tube current modulation with quality reference value 140 mAs, iterative reconstruction iteration strength 3, reconstruction filter/kernel soft tissue, primary reconstruction slice thickness/increment 3/3 mm and multiplanar reconstruction slice/increment 3/3 mm. 

Pretreatment bone densities were measured by one radiologist (K.N.) with 6 years of CT reading experience; measurements were performed on CT Bone Reading software (syngo.via, version VB60; Siemens Healthineers, Erlangen, Germany). Bone density was measured in Hounsfield units (HU) in the center of the L1 vertebral body with a region of interest of 4 cm^2^.

All available tomography follow-up examinations were reviewed. Available scans were from different modalities (CT, MRI and PET/CT), different scanners and different qualities. In reviewing cross-sectional scans, we proceeded as follows. First, in each subject the radiation treatment plan was checked to determine the upper level of the radiation field. Then, we searched for radiation-related side effects in bone, such as pelvic insufficiency fractures, osteonecrosis and bone malignancies. The types of bone affections and their localizations were recorded. In the case of multiple affections, all were taken in consideration. Bone pathologies outside the radiation field were not considered as radiation related. All scans were independently reviewed by 2 radiologists with 26 and 6 years of CT reading experience (H.M. and K.N.); their records then were compared, and in the case of discrepancies, a third opinion on the radiology board was asked.

### 2.3. Statistics

Analyses were performed using STATISTICA software version 12 (Tibco Software Inc., Palo Alto, CA, USA). First, data were tested for statistical distribution; all collected data were normally (parametrically) distributed. We report descriptive statistics as mean ± standard deviation. Data of both groups and subgroups were compared by paired *t*-test, x^2^ test and the Fisher test. For correlation between variables, the Pearson test was used; *p*-values < 0.05 were considered as statistically significant. 

## 3. Results

### 3.1. Patient Selection Data

We selected 190 female patients. Sixty-three subjects were excluded due to a lack of cross-sectional imaging follow-up. No one was included due to previous or current trauma in history, long-lasting corticosteroid therapy or previous osteoporosis treatment. Thus, 127 subjects were included: 63 patients in the CC group (treated for cervical cancer) and 64 patients in the EC group (treated for endometrial cancer). 

All patients in the CC group were treated by radical RT with EBRT by intensity-modulated radiation therapy (IMRT), typically at a dose of 45 Gy/25 fractions. Sixty subjects (95.2%) underwent intracavital BRT guided by 3D imaging with doses of 4 × 6.5 Gy. Fifty-nine subjects (93.7%) underwent concomitant chemotherapy by cisplatin, five applications once weekly from day 1, with a dose of 40 mg/m^2^ to an absolute dose of 70 mg. Moreover, 46 patients (73.0%) underwent lymph node RT at variable doses. 

All patients in the EC group underwent pelvic EBRT by IMRT, typically at a dose of 45 Gy/25 fractions, and vaginal BRT with doses varying from 2 × 5 Gy to 3 × 5 Gy. Eight subjects (12.5%) underwent lymph node RT at variable doses. Twenty-four females (37.5%) were treated by adjuvant paclitaxel and carboplatin chemotherapy. 

Both groups significantly differed in the total RT dose received. The mean total RT dose in the CC group was 80.0 ± 14.1 Gy, versus 62.1 ± 13.9 Gy in the EC group: *p* < 0.001. The mean time of cross-sectional imaging follow-up in the CC group was 32.8 ± 15.6 months, while the subjects in the EC group were followed at a mean of 26.0 ± 18.1 months: *p* = 0.055. However, patients in the CC group underwent significantly more imaging examinations. The mean number of imaging examinations in the CC group was 9.2 ± 4.7, versus 4.5 ± 2.4 in the EC group: *p* < 0.001. 

The CC group subjects were significantly younger than the subjects in the EC group: 52.8 ± 13.1 years vs. 66.0 ± 9.9 years (*p* < 0.001). Both groups also significantly differed in BMI; in the CC group, the mean BMI was 24.9 ± 5.9 kg/m^2^, versus 30.3 ± 6.8 kg/m^2^ in the EC group (*p* < 0.001). Significant differences were found in the age of menopause: 45.5 ± 7.9 years in the CC group versus 52.0 ± 4.4 years in the EC group (*p* < 0.001). Ten of sixty-three patients in the CC group were treated by hormonal substitution, versus one of sixty-four subjects in the EC group (*p* = 0.004). For more detail, see Table 1.

### 3.2. Cross-Sectional Imaging Analysis, Pretreatment Bone Density Measurement and Statistical Analyses

Generally, we did not find any significant differences between both groups with regard to the number of subjects suffering from radiation-related bone side effects. In the CC group, 18 subjects (28.6%) suffered from radiation-related bone side effects; in the EC group, radiation-related bone side effects were found in 17 subjects (26.6%; *p* = 0.8). No bone malignancies were detected. Osteonecrosis was present only in two subjects in the EC group; in both cases, osteonecrosis was situated in the femoral head. The most common bone side effects in both groups were sacral insufficiency fractures. In the CC group, we found 15 (23.8%) cases of sacral insufficiency fractures: a unilateral sacral fracture was present in 8 cases, bilateral fractures were present in 7 cases, and in 2 subjects, sacral fractures were combined with other fractures such as a pubic fracture or an L5 vertebral compression fracture. In the EC group, sacral insufficiency fractures were found in 12 (18.8%) cases, in 5 subjects unilateral and in 7 females bilateral; in 3 females, sacral fractures were found in combination with other fractures such as a pubic fracture and/or an L5 vertebral compression fracture. See also Table 2.

**Figure 1:**
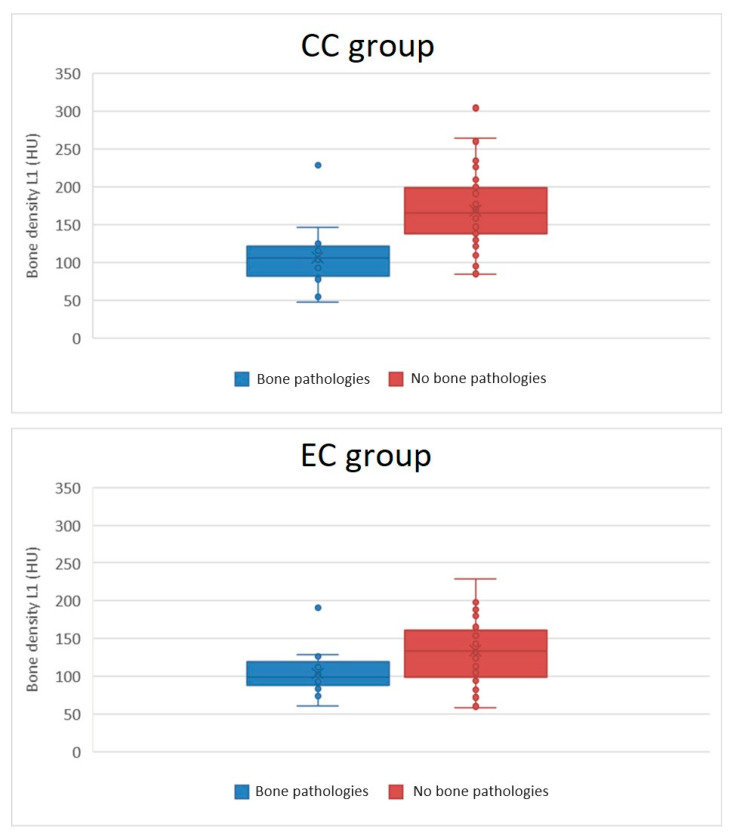
Graph of relation between radiation-related bone side effects and bone densities.

**Figure 2:**
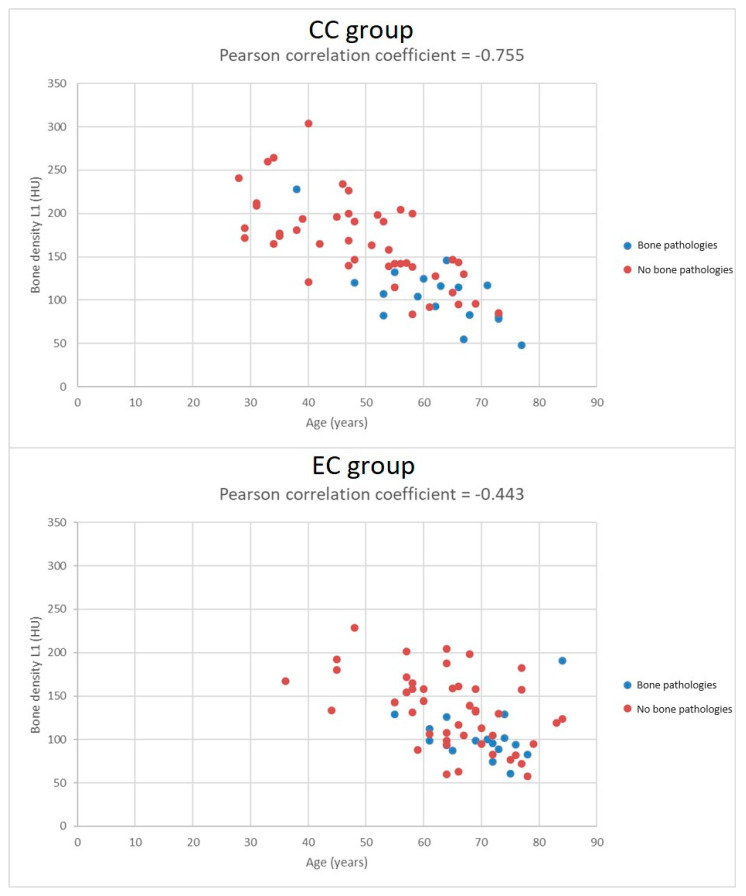
Graph of correlation between bone density and the age of treated females.

Subjects in the CC and EC groups significantly differed in pretreatment CT bone density of the L1 vertebral body. The mean bone density in the CC group was 150.5 ± 54.5 HU, while in the EC group it was 125.8 ± 41.2 HU: *p* = 0.005. In the CC group, bone density strongly correlated with subject age (r = −0.755); in the EC group, only a weak correlation between age and bone density was apparent (r = −0.443). See also Figure 1 and Figure 2. There were no correlations between pretreatment bone density and BMI (r = 0.096 and r = 0.247, respectively).

We found significant differences when we divided both groups into subgroups according to the presence of radiation-related bone side effects. In the CC group, subjects with radiation-related bone pathologies were significantly older (mean 62.4 ± 10.1 vs. 49.0 ± 12.4 years, *p* < 0.001), and their pretreatment bone density was significantly lower (106.3 ± 40.0 vs. 168.2 ± 49.5 HU, *p* < 0.001). We did not find any remarkable differences in BMI and in the total RT dose received. For more detail, see Table 3. 

In the EC group, patients with radiation-related bone pathologies were older (mean 70.0 ± 7.3 vs. 64.6 ± 10.4 years); however, the difference was not significant (*p* = 0.057). Pretreatment bone density in EC group subjects with bone pathology was significantly lower (103.8 ± 29.0 vs. 133.8 ± 42.3 HU, *p* = 0.009), and also their BMI was significantly lower (mean 26.1 ± 4.9 vs. 31.8 ± 6.9 kg/m^2^, *p* = 0.003). The total RT dose received in both subgroups of the EC group with/without bone pathology was nearly identical. For more detail, see Table 3.

Finally, we compared subjects that suffered from radiation-related side effects from both groups. Subjects in the CC group with bone pathologies were significantly younger than subjects from the EC group (mean 62.4 ± 10.1 vs. 70.0 ± 7.3 years, *p* = 0.018); CC subjects with bone pathologies received a significantly higher total RT dose (77.9 ± 5.8 vs. 62.2 ± 11.4 Gy, *p* < 0.001) than EC subjects with bone pathologies. We did not find any correlation between fracture occurrence and total radiation dose. We did not find significant differences in bone density or BMI. Bone densities in both subgroups with bone pathologies were similarly low (106.3 ± 40.0 vs. 103.8 ± 29.0 HU, *p* = 0.829), similar to their BMI (23.8 ± 4.2 vs. 26.1 ± 4.9 kg/m ^2^, *p* = 0.141). However, there was no correlation between bone density and BMI. For more detail, see Table 3 and Figure 1 and Figure 2. 

## 4. Discussion

Despite the fact that both cohorts of subjects significantly differed in most pretreatment and treatment variables, radiation-related fractures were detected in 29% and 27% of treated females. Females treated for cervical cancer were a mean 13 years younger, their BMI was 5.4 kg/m^2^ lower, they achieved menopause at a younger age, they received an about 18 Gy higher total RT dose, and most received concomitant chemotherapy with cisplatin. Females that suffered from fractures in both cohorts had significantly and comparably lower L1 vertebral body density (mean 106.3 ± 40.0 and 103.8 ± 29.0 HU, respectively) and had similarly low and comparable BMIs (mean 23.8 ± 4.2 vs. 26.1 ± 4.9 kg/m^2^). However, subjects treated for cervical cancer without bone pathologies had similarly low BMIs; therefore, that finding only mirrored group differences, and no correlation between bone density and BMI was found. Nevertheless, females treated for cervical cancer with fractures were significantly younger than females with fractures treated for endometrial cancer (mean 62.4 ± 10.1 vs. 70.0 ± 7.3 years). However, bone density strongly correlated with age only in the cohort treated for cervical cancer. Females treated for cervical cancer with fractures received a significantly higher total radiation dose (mean 77.9 ± 5.8 vs. 62.2 ± 11.4 Gy); however, those findings again only mirrored group differences. 

Bone marrow is highly radiation-sensitive tissue. Free-radical formations likely induce bone vasculature injury and mesenchymal stem cell microenvironment damage [18]. Bone marrow in the radiation field undergoes fatty replacement, and RT leads to a significant decline in bone density [19]. Therefore, radiation-related bone side effects such as fractures are not rare occurrences, especially in weight-bearing regions such as the pelvis or femoral head. There are a number of studies that have documented pelvic insufficiency fractures after radical RT for cervical cancer or pelvic cancers [14,16,17]; however, radiation-related fractures exclusively affecting females treated for endometrial cancer have received little interest. According to a meta-analysis by Chung et al. (2021) [16], the pooled prevalence of pelvic insufficiency fractures associated with radical RT for cervical cancer was estimated at 14%. In our study, the rate of pelvic fractures was higher, with 29% of patients suffering fractures. According to published data, pelvic insufficiency fractures after RT are significantly associated with higher age, which is considered to be a strong predisposing factor [16,20]. Our results are partly in concordance with those data; in both cohorts, females that suffered from pelvic fractures were older, but the difference was significant only in the cohort of subjects with cervical cancer. Moreover, patients with fractures treated for cervical cancer were significantly younger than those treated for endometrial cancer.

Lower BMI, postmenopausal age and pretreatment osteoporosis are considered as further risk factors for radiation-related fractures [17,20,21,22,23]. In our study, the whole group of patients treated for cervical cancer had significantly lower BMIs than the group of patients with endometrial cancer; moreover, their BMIs were low compared to subjects with fractures that were treated for endometrial cancer. Due to the retrospective design of the study, there were no available data with respect to osteoporosis, such as dual-energy absorptiometry (DXA) or serum biomarker levels. Therefore, we retrospectively measured bone density in the L1 vertebral body on pretreatment CT. According to a study by Abbouchie et al. (2022), L1 bone densities measured on CT correlate with L1 DXA values, and values >180 HU rule out osteoporosis with high certainty [24]. From our data, we can state that subjects with radiation-related fractures in both cohorts suffered from obvious osteoporosis; their mean L1 bone density was 106.3 ± 40.0 and 103.8 ± 29.0 HU, respectively. We found strong correlations between pretreatment bone density and age only in patients treated for cervical cancer. It is necessary to mention that the whole group of subjects treated for cervical cancers was significantly younger, and also those with fractures were significantly younger in comparison to subjects treated for endometrial cancer. Surprisingly, we did not find a correlation between bone density and BMI. 

The groups significantly differed in the total RT dose and chemotherapy. A majority of patients with cervical cancer were treated by concomitant chemotherapy by cisplatin. In contrast, only a minority of subjects treated for endometrial cancer received adjuvant chemotherapy. It remains unclear how RT dose, different RT modalities and RT volume may be related to the risk of radiation-related bone side effects [25]. In our study, there was no correlation between fracture occurrence and total RT dose. Some studies have suggested that higher RT doses are associated with higher rates of insufficiency fractures [26,27,28]. According to an observational cohort study by Vitzthum et al. that involved 28,354 patients who underwent RT of pelvic cancers, IMRT and BRT alone reduced the risk of pelvic insufficiency fractures; however, this was only in older women [20]. A meta-analysis by Sapienza et al. (2020) showed similar results. They analyzed 21 studies and concluded that the use of IMRT was associated with a lower fracture rate [29]. In our study, all patients underwent IMRT, and we can clearly state that a higher RT dose was not associated with a higher rate of radiation-related bone side effects. 

It was proven that concurrent cisplatin-based chemotherapy plus RT improves overall survival in females treated for locally advanced cervical cancer [30,31]. However, according to some studies, chemotherapy potentiates the risk of bone toxicities after radical RT [21,32]. In a study by Misra et al., bone toxicities were documented in 25% of females treated for cervical cancer by RT and concomitant chemotherapy, versus 16% of patients who underwent only radical RT [32]. In our study, the rate of radiation-related fractures was similar in both groups, although nearly all of the patients with cervical cancer were treated by concomitant chemotherapy, and only a minority of patients treated for endometrial cancer received adjuvant chemotherapy.

Our study has several important limitations. The most important limitation of the present study is its retrospective, single-center design and limited sample size. The limited sample size and selection bias we consider as the most important limitation. In the selected time period, one-third (63) of subjects did not undergo cross-sectional follow-up imaging and were excluded from the study; those patients may have affected our results. However, according to recommendations, regular cross-sectional imaging follow-up such as CT, MRI or PET/CT is not generally recommended [2,4,6]. Moreover, the available cross-sectional imaging scans were of different modalities, were conducted with different scanners and often had different qualities and were without temporal regularities. In addition, variability in timing and the number of scans between the longer follow-up could affect fracture identification.

Despite the abovementioned limitations, we have shown that radiation-related bone side effects were strongly associated with low CT bone density of the L1 vertebral body, reflecting osteoporosis. However, we honestly state that CT bone density measurements cannot replace an osteoporosis assessment by DXA. On the other hand, radiation-related bone side effects could affect patient quality of life [7]. Our study suggests a simple way to search for patients at risk, by measurement of L1 bone density on CT. The method is straightforward and without additional cost. In patients at risk, especially in young patients, preventive treatment may be considered. For future research, we suggest prospective studies evaluating preventive osteoporosis treatment in at risk patients.

## 5. Conclusions

Despite the fact that subjects treated for cervical and endometrial cancer significantly differed in most pretreatment and treatment variables, the rate of radiation-related fractures was similarly high, at 29% and 27%, respectively. Fractures in both groups were strongly associated with low pretreatment bone density of the L1 vertebral body, indicating osteoporosis, which is an obvious risk factor for radiation-related bone side effects. L1 bone density measurement is an easy way to search for patients at risk; in those patients, preventive treatment should be considered. Surprisingly, no correlation between bone density and BMI was found, and age correlated with bone density only in patients treated for cervical cancer.

## Figures and Tables

**Table 1:** Patient selection data.

	CC Group N = 63	EC Group N = 64	*p*-Value
Age (years)	52.8 ± 13.2	66.0 ± 9.9	<0.001
Lymph node RT (N of subjects)	46	8	<0.001
BRT (N of subjects)	60 (intracavitary)	62 (vaginal)	0.635
Total RT dose (Gy)	80.0 ± 14.1	62.1 ± 13.9	<0.001
Hormonal substitution (N of subjects)	10	1	0.004
Concomitant chemotherapy (N of subjects)	59	0	<0.001
Adjuvant chemotherapy (N of subjects)	0	24	<0.001
Radical surgery	0	64	<0.001
Menopause (years)	45.4 ± 7.9	52.0 ± 4.4	<0.001
BMI (kg/m^2^)	24.9 ± 5.9	30.3 ± 6.8	<0.001
Time of imaging follow-up (months)	32.8 ± 15.6	26.0 ± 18.1	0.055
N of imaging exams	9.2 ± 4.7	4.5 ± 2.4	<0.001

CC, patients treated for cervical cancer; EC, patients treated for endometrial cancer; BRT, brachytherapy; RT, radiotherapy; N, number; BMI, body mass index.

**Table 2:** Radiation-related bone side effects detected on imaging scans.

	CC Group	EC Group	*p*-Value
Osteonecrosis (N of subjects)	0	2	0.496
PIF (N of subjects)	18	15	0.613
Sacral PIF (N of subjects)	15	12	0.574
Pubic PIF (N of subjects)	1	2	0.577
Vertebral compression fracture (N of subjects)	3	5	0.506

CC, patients treated for cervical cancer; EC, patients treated for endometrial cancer; PIF, pelvic insufficiency fracture; N, number.

**Table 3:** Differences related to radiation-related side effects.

**A**			
**CC Group**	**Subgroup with Fractures**	**Subgroup without Fractures**	***p*-Value**
N	18	45	-
Age (years)	62.4 ± 10.1	49. 0 ± 12.4	<0.001
Density (HU)	106.3 ± 40.0	168.2 ± 49.5	<0.001
BMI (kg/m^2^)	23.8 ± 4.2	25.4 ± 6.5	0.329
Total RT dose (Gy)	77.9 ± 5.8	80.6 ± 16.2	0.489
**B**			
**EC Group**	**Subgroup with Fractures**	**Subgroup without Fractures**	***p*-Value**
N	17	47	-
Age (years)	70.0 ± 7.3	64.6 ± 10.4	0.057
Density (HU)	103.8 ± 29.0	133.8 ± 42.3	0.009
BMI (kg/m^2^)	26.1 ± 4.9	31.8 ± 6.9	0.003
Total RT dose (Gy)	62.2 ± 11.4	62.1 ± 14.7	0.966
**C**			
	**CC** **Subgroup with Fractures**	**EC** **Subgroup with Fractures**	***p*** **-Value**
N	18	17	-
Age (years)	62.4 ± 10.1	70.0 ± 7.3	0.018
Density (HU)	106.3 ± 40.0	103.8 ± 29.0	0.829
BMI (kg/m^2^)	23.8 ± 4.2	26.1 ± 4.9	0.141
Total RT dose (Gy)	77.9 ± 5.8	62.2 ± 11.4	<0.001

CC, patients treated for cervical cancer; EC, patients treated for endometrial cancer; N, number of subjects; BMI, body mass index; RT, radiotherapy.

## Data Availability

Data are available upon a reasonable request to the study investigators.
